# “It’s gotta be done right way”: a qualitative study exploring the acceptability of a proposed longitudinal cohort study of young Aboriginal children in Alice Springs

**DOI:** 10.1186/s12913-023-10148-9

**Published:** 2023-11-06

**Authors:** Catherine Lloyd-Johnsen, Angela Hampton, Emma Stubbs, Sam Moore, Sandra Eades, Anita D’Aprano, Sharon Goldfeld

**Affiliations:** 1 Centre for Community Child Health, Murdoch Children’s Research Institute, Melbourne, VIC Australia; 2 Department of Paediatrics, University of Melbourne, Melbourne, VIC Australia; 3 Central Australian Aboriginal Congress, Alice Springs, NT Australia; 4 Menzies School of Health Research, Darwin, NT Australia; 5 Flinders University, Adelaide, SA Australia; 6 Melbourne School of Population and Global Health, The University of Melbourne, Melbourne, Australia; 7 Royal Children’s Hospital, Melbourne, VIC Australia

**Keywords:** Australian Aboriginal, Indigenous, Child Health, Longitudinal studies, Qualitative study, Thematic Analysis

## Abstract

There is a need for quality longitudinal data on the health and well-being of young Aboriginal and Torres Strait Islander children (hereafter Aboriginal) in Alice Springs that can be used for research, planning and evaluation. The primary aim of this descriptive qualitative study was to determine whether or not a proposed cohort study would be acceptable to the local community. The proposed cohort study will prospectively examine various factors, events and exposures in early life that give Aboriginal children the best chance to grow up strong and lead a healthy happy life. Decisions on specific priority issues to be addressed and study procedures will be determined by local Aboriginal researchers and community members during a future co-design phase. 27 semi-structured interviews and 3 focus group discussions (FGD) were conducted with a range of community stakeholders and parents/caregivers of young Aboriginal children from Alice Springs in the Northern Territory (NT) of Australia. Audio recorded data were transcribed and imported into NVivo12 qualitative software for reflexive thematic analysis. Three major themes concerning acceptability of the concept were constructed from the analysis: (1) Have to be mindful, (2) Duplication of data, and (3) “It’s gotta be done right way”. There was general support for the concept, however, many participants felt that a cautious and slow approach was necessary. Recommendations included focusing on building trust, taking it slow, and ensuring the study is conducted by local Aboriginal researchers. Barriers to feasibility noted included the high mobility of families, competing demands, and privacy concerns. Findings from this qualitative study support the feasibility and acceptability of a future cohort study of young Aboriginal children in Alice Springs. Leadership from respected local Aboriginal researchers and key stakeholders will be critical to its success.

**Supplementary Information:**

The online version contains supplementary material available at 10.1186/s12913-023-10148-9.

## Background

Using a life course approach, longitudinal cohort studies can identify critical periods of growth and development in gestation, early childhood, and adolescence that affect long-term health and wellbeing [1–3]. They can help us better understand complex interactions that drive inequities affecting the health of Aboriginal and Torres Strait Islander children. These inequities are not random, but systematic and strongly influenced by the social determinants of health. Hereafter we use the term ‘Aboriginal’. As a stand-alone term, we acknowledge this is not inclusive of Torres Strait Islander peoples. However, the term better represents the Central Australian population, and Aboriginal people in Central Australia prefer to identify this way [4].

It is undeniably expensive to collect longitudinal data. Prospective longitudinal cohort studies collect repeated measures on the same group of individuals over a defined period of time [5]. These studies require a significant commitment from participants and are generally more resource intensive than other types of epidemiological studies [5, 6]. Numerous factors can affect the successful implementation of a cohort study, such as attrition, which occurs when participants drop out. Thus, it is imperative to determine feasibility as early as possible. Feasibility studies play a key role in refining the design of proposed research studies. They are used to understand how things will be implemented in a future stage of the research. This can include the evaluation of recruitment, retention, data collection procedures and analysis plans [7]. Another important facet is determining ‘acceptability’. This refers to the study, and its procedures, being perceived by participants as “agreeable or satisfactory” [8]. In the Aboriginal context there are additional considerations; it is essential that the proposed cohort study is acceptable and appropriate to meet the cultural needs of the community [9].

Investing in longitudinal research that focuses on these early years has the greatest potential to reduce health disparities and shift trajectories toward improved health outcomes for children. A recent systematic review of longitudinal studies focusing on the health and wellbeing of Indigenous children globally found 77 individual longitudinal studies including Australian Aboriginal and Torres Strait Islander children (0–18 years of age). While this was the highest number of studies for any nation, only a handful included the recruitment of Aboriginal children from Central Australia [10].

During a research planning meeting in 2013, the Central Australian Aboriginal Congress (Congress) in Alice Springs called for more research to focus on the health and wellbeing of young Aboriginal children as they grow [11]. This request was the genesis for a partnership proposal, between Congress and researchers from the Murdoch Children’s Research Institute (MCRI), to establish a longitudinal cohort study of young Aboriginal children in Alice Springs. The idea was that the study would help the service and its research partners better understand how, why, and when kids in Alice Springs start along a path to better or worse health outcomes. The resulting data could be used by the community to make decisions that might help kids be healthier as they grow and develop. The proposed cohort study would likely entail collecting a variety of health and lifestyle information gathered using multiple data collection methods, including direct face-to-face assessments and the low burden use of existing administrative data.

Before plans progressed further, it was deemed important to first determine feasibility and acceptability. Thus, the primary objective of this study was to explore community acceptance of, and support for, a proposed longitudinal cohort study focusing on the health and wellbeing of local Aboriginal children (0–12 years of age) in and around Alice Springs (NT). In addition, we aimed to explore parents’, caregivers’ and community stakeholders’ perspectives on the feasibility of the proposed cohort study including their attitudes regarding data use and privacy. It is intended that these findings will facilitate the refinement of a future proposal to be presented to the community, whose support will ultimately decide priority issues to be addressed and whether it should proceed or not.

## Methods

### Study Design

This descriptive qualitative study is part of a program of research conducted in partnership between Congress and the Murdoch Children’s Research Institute (MCRI) as part of the *Atyepe-atyepe Iwerre Ampe-ke* [Healthy Journey for Kids] Feasibility Study.

### Sampling and recruitment

Study participants were parents and caregivers of young Aboriginal children and key community stakeholders who routinely work with local Aboriginal families across a variety of different sectors, including health, education, and community organisations. A combination of purposive and snowball sampling was used to identify potential participants. Our recruitment catchment area included the township of Alice Springs and up to a 100 km radius, in line with Congress’ own definition of ‘town’ as opposed to Congress auspiced remote service areas.

We purposively sampled individuals according to their role across the different settings to obtain maximum diversity. Our goal was to recruit at least two individuals from each of the sectors identified as important (health, education and other service providers) to young Aboriginal families. This was to ensure a wide range of experiences and participant perspectives were included in the sample [12, 13]. We started interviewing a number of key stakeholders and utilised their networks and recommendations to identify who to interview next. Our Congress partners were also asked to verify the completeness of our list, mapping key community stakeholders.

The Congress Research Manager initially contacted nominated stakeholders to invite them to participate. Once potential participants agreed to be contacted, the Information Statement & Consent Form were sent out via email with an invitation to discuss participation with a research team member over the phone. The consent process used was intentionally flexible to allow sufficient time for potential participants to discuss the study with the researchers on multiple occasions. Following obtaining written informed consent, a mutually convenient time and venue for the interview were agreed to. COVID-19 restrictions meant that some of the stakeholder interviews were conducted via the video conferencing software Zoom. All parents and caregivers were recruited and interviewed in-person by one of the three local authors (AH, ES or SM).

To be eligible, participants needed to be: a parent/ caregiver of a young Aboriginal child (0–12 years of age) residing in the geographical area in and around Alice Springs serviced by Congress; or a community stakeholder who had experience working with, or supporting, young Aboriginal families. Eligible participants themselves were residents of Central Australia, at least ≥ 18 years of age, able to speak English, and provided consent to have their interview recorded.

We set out to recruit a 1:1 ratio of parents/caregivers to community stakeholders up to a sample of between 15 and 30 individuals. This was reported as a common sample size in other qualitative studies looking to identify patterns across data [14]. Ultimately, recruitment continued until we felt that the right mix of participants were included and that we had enough data to tell “a rich, complex and multi-faceted story” [15].

### Data collection

An interview guide was developed in response to a literature review on the feasibility of longitudinal research involving Indigenous children. The lead Aboriginal Investigator (SE) provided initial input into the language of the first draft of the interview and FGD guides developed by the first author (CLJ). The proposed guides were then further reviewed by each of the remaining authors (SG, AD, SM, AH & ES). Initial pilot testing was conducted with an external non-Aboriginal researcher, and later with an external Aboriginal researcher with extensive experience conducting qualitative data collection with Aboriginal families in community settings.

Both guides included open-ended questions to encourage participants to express their ideas in their own words. Interviews were kept flexible and reactive to participants’ responses—including both verbal and body language. If the interviewer read the participant’s body language to be closed off or disengaged, questions were asked in a different way. Prompts were used to elaborate on participants’ responses and topics were tailored slightly to reflect their specific involvement with young Aboriginal children as either parents, caregivers or community stakeholders.

The team responsible for conducting fieldwork had prior experience in qualitative research. It was considered important to allow time for silence after each question. This gave participants time to think about the question before responding [4]. Fieldwork was carried out by two female Aboriginal researchers (AH & ES), a local male non-Aboriginal researcher (SM) and a female non-Aboriginal interstate researcher (CLJ). Culturally based gender sensitivities were considered when conducting interviews with male and female participants as directed by the Lead Aboriginal Cultural Advisor at Congress. Data collection occurred over a 14-month period from 2020 to 2021. Participants received a $20 gift card as reimbursement for participation. The interview guides used can be found in Additional file 1.

### Data analysis

Data analysis was undertaken according to Braun & Clarke’s (2006) six phases of thematic analysis [16]. Digital voice recorders were used to record the interviews and FGDs. Audio recordings were manually transcribed verbatim and cross-checked by two authors (SM & CLJ) for accuracy. The transcripts were then anonymised and imported into qualitative data analysis software (NVivo Version 12, 2012, QSR; International Pty Ltd. Melbourne, Australia). Two authors (CLJ & SM) independently read and re-read each of the transcripts to become familiar with the data in Phase 1. Individual reflections and brief memos were drafted.

The overall data analysis was led by the first author (CLJ), using a bottom-up approach to code text segments in each transcript into preliminary nodes. Both semantic and latent features of the data were examined in this second phase. One author (SM) coded a subset of 10 transcripts, whilst two other authors (AH & ES) reviewed at least one transcript each independently. The purpose of this exercise was not to establish inter-rater reliability, as all coding was led by the first author (CLJ). Instead, we aimed to produce a collaborative approach to data interpretation where we compared and reflected on our different perspectives as a team. Using this approach, we were able to develop a more nuanced and richer understanding of the data, rather than simply working toward consensus (Braun & Clarke 2019). The bottom-up approach used in Phase 2 coding allowed the data to drive the formulation of themes, rather than predefining them. The first author (CLJ) reviewed all the transcripts coded by the team to determine whether there were any other relevant themes that needed to be added. This process helped contextualise comments made by the team. The first iteration of nodes produced a number of finely grained themes (Phase 3). Three authors (SM, AH & ES) read all the data under each node and discussed preliminary themes that the first author (CLJ) identified.

Collaboratively, we worked to refine, classify, and synthesise candidate themes (Phase 4). We reflected on our different experiences and positions and how this may have shaped our interpretation of the data. We have addressed this more comprehensively in the Position Statement in Additional File 2. Five candidate themes were initially identified. The preliminary thematic map was further discussed until we all agreed that the five themes should be collapsed into three major themes with some sub-themes. The final theme names assigned convey shared meaning across the dataset (Phase 5). Our research is set within a social constructionist perspective, which asserts that people interpret and make sense of their experiences based on their social, political, and historical contexts [17, 18]. Through the writing process (Phase 6), we reviewed and refined the themes and sub-themes. In framing the final results (Phase 6) we adopted a strengths-based approach [19]. All authors contributed to the final manuscript. The senior Aboriginal researcher (SE) and the two local Aboriginal researchers (AH & ES) provided cultural guidance on the interpretation of evidence.

## Results

Twenty-seven interviews and three FGDs were with a total of 42 participants (Table 1). The size of FGDs ranged from 3 to 5 participants each. On average interviews lasted 41 min (range 14–68 min) and focus group discussions lasted 83 min (range 65–97 min). There were 36 female and 6 male participants in this study (Table 1). Sixteen were recruited as parents/caregivers of young Aboriginal children and 26 were recruited in their role as various community stakeholders as health or education professionals. Fifty-seven per cent of the sample (n = 24) self-identified as Aboriginal. Many of the Aboriginal stakeholders described dual roles in their responsibility as carers of their own children, grandchildren or extended kin in the community. The use of word of mouth and pre-existing networks was the most successful strategy for the recruitment of parents/caregivers. This ‘snowball’ approach has been previously found to work well in close knit Aboriginal communities [20]. Our sample included a broad range of ages, gender and occupational status. We interviewed parents, aunties, uncles, foster/kinship carers and grandparents ranging in age from 23 to 70 + years. Several Central Australian Aboriginal language and kinship groups were represented in our sample. To maintain anonymity, we have changed the names and identifying characteristics of study participants including the names of people, locations and workplaces they spoke about. We have reported general demographic information alongside quotes including their occupation/role and gender.

**Table 1:** Participant Characteristics

Participant Characteristics		
	Number	Percentage (%)
Gender		
Female	36	85.7
Male	6	14.3
Aboriginal		
Yes	24	57.1
No	18	42.9
Role		
Parent/Caregiver	16	38.1
Stakeholder/ Community representative	26	61.9

Three themes were constructed: (1) Have to be mindful, (2) Duplication of data, and (3) “It’s gotta be done right way”.

### Theme 1: have to be mindful

Most participants were positive about the establishment of a longitudinal study of young Aboriginal children and indicated acceptability:

*“We want to know, are we making an impact... and is our model working for the families… the longitudinal stuff is really important, that’s always been a concern for me…I wanna know, ‘Are we doing the right thing?”* (Female Aboriginal Child & Family Service Provider).

When asked directly, most participants interviewed reported hypothetical willingness for the children in their lives to participate in a longitudinal study. Whilst most were positive there were several recurring caveats that are explored in the following three subthemes titled: (a) Be mindful of confidentiality & worries about shame or guilt, (b) Be mindful that the community is over researched & over serviced, and (c) Transient population.

### Subtheme (a) be mindful of confidentiality & worries about shame or guilt

Confidentiality was raised as a key concern. Some of the participants expressed confidence in the researcher’s ability to protect their privacy, whilst others were more cautious. Negative experiences around past use of data were cited:

*“[It’s] a real fear… for our mob… if I share too much information or if they see something within my home, I’m going to get reported and… that’s just a fear that’s really embedded in our communities*” (Female Aboriginal Parent/Caregiver).

Reference to being judged or experiencing feelings of shame from study outcomes was also brought up as a potential barrier to uptake. The reluctance to take part was described as being contingent on how families were coping in general. Recommendations provided by participants on how to mitigate these concerns included: providing clear information about exactly what data will be used or collected, and for local Aboriginal researchers to obtain specific informed consent from families.

## Subtheme (b): be mindful that the community is over researched & over serviced

Another key concern raised about acceptability was the need to be mindful of not further burdening the community. Despite the strong positive response to the proposed concept, nearly every participant referred to the fact that Aboriginal people are the most researched people on the planet. The desire for research to be action orientated was evident. Data duplication and research without translation was perceived as pestering already overburdened Aboriginal families. One participant described this as being like “*parasites in parachutes*”. Past negative experiences of fly-in fly-out researchers offering little benefit to the communities were discussed:

“*People come and do research on our kids… just to… parachute in and then disappear, they don’t engage with us properly, and then we get no feedback about the outcomes… there’s no engagement… moving forward [how] that research [should] inform programs or health systems”* (Female Non-Aboriginal Health Professional).

These past experiences continue to influence local attitudes towards research. Participants were adamant that if the research is to be meaningful it must have tangible benefits for the people. This need for research to be action orientated and directly benefit families and the wider community is further explored in Theme 3.

### Subtheme (c) transient population

The push and pull factors that see Aboriginal families move between Alice Springs and their surrounding homelands is a well understood fact of life for many in Central Australia. Stakeholders talked of how these movements can sometimes affect timely access to health and educational services. Participants also underscored the potential challenges the proposed longitudinal study could expect in trying to maintain contact with families over time, citing that it was common for individuals to change phone numbers regularly, live in areas without mobile reception or have limited access to the internet. Despite these logistic challenges, it was suggested that the proposed research should factor mobility into its design.

On the whole, mobility was described as a positive opportunity for children to remain connected to culture and country which is an important protective factor for their social and emotional wellbeing. Participants talked about how mobility was a fundamental strength for young Aboriginal families in Central Australia:

*“For many Aboriginal children… their cultural ceremonies are still really important…And they will… go out bush for a certain amount of time then come back into town” (Female non-Aboriginal Educator).*

### Theme 2: duplication of data

Research duplication was an important issue, as was the duplication of data collection across local health and education services too. Participants reported that local Aboriginal families are often required to retell their “story” over and over again when accessing services and yet the data is kept in “silos”. The tension between needing to preserve confidentiality and the desire to reduce burden on families when collecting data was cited by several stakeholders interviewed.

Sharing data between fundamental health and development services was perceived as an insurmountable barrier, indicating salient challenges for a research project. It’s not only the fact that families must retell their story to multiple organisations but also over time to different individuals within teams as funding and staff turnover is yet another constraint. One parent described themselves, and others, as being:

*“sick of having to tell their story over and over and over… it’s just hard”* (Female Aboriginal Parent/Caregiver).

These issues were perceived to likely affect families’ willingness to engage with longitudinal research.

### Theme 3: “It’s gotta be done right way”

All participants were explicit about how the proposed study should be conducted. Robust language was used to demonstrate what the proposed research must do in order for it to be acceptable to the local community.

From this data, we generated Theme 3: “It’s gotta be done right way”. Within this theme we identified four prominent subthemes: (1) “In the hands of the people”, (2) Build trust & slow it down, (3) Tangible benefits for all, and (4) Oral stories are important too.

### Subtheme (a) “In the hands of the people”

Throughout the data it was clear that participants felt the proposed research must be led by local Aboriginal people. To be acceptable, participants expressed the need for community ownership. One participant asserted the need for genuine co-design of the proposed research:

*“It needs to be… co-designed … genuinely done, and that’s not [just] a reference group, that is Aboriginal control and leadership and ownership at every single level, including Aboriginal people or a representative group should be approving who uses what data…for what purpose”* (Female non-Aboriginal NGO worker).

A clear need for Aboriginal staff to be conducting the research was conveyed by all. Linking success to the employment of local Aboriginal people was described by one stakeholder as the only guarantee of continuity. Several parents and caregivers talked of feeling more at ease working with local Aboriginal researchers. Language barriers were also noted as something that might impact families’ willingness to take part in the proposed study. This concern would be negated if the researcher was a local Aboriginal person. Parents talked about their different experiences interacting with Aboriginal versus non-Aboriginal researchers. One participant further explained why it has to be local people conducting the interviews and collecting the data from families because of a shared experience of life in Central Australia:

*“Cause we can all relate to these things when we’re talking about it… it’s like, how do you explain that to other people? There’s so much going on in this way of life that you mob* [meaning non-Aboriginal people] *just don’t know”* (Female Aboriginal NGO Worker).

Partnerships with local Aboriginal controlled organisations was recognised as an important contributor to acceptability. Another important aspect of acceptability identified was the need for the study to be culturally responsive and specific to the needs of the local community. It was clear that actively listening to community members who are the experts is imperative. Participants recommended that local Aboriginal researchers make sure that any questions to be asked of families are culturally responsive and not too intrusive, potentially causing harm or shame. It was also noted that the study should be conscious of gender sensitivities.

### Subtheme (b) build trust & slow it down

Building trust was perceived as another crucial factor to acceptability. Participants felt strongly about the need to build trust. Having the right approach when going into community was important. Getting key local people involved in the engagement with families was another common suggestion to increase participation.

We also found that giving ample time to the process of community engagement and informed consent was also very important to participants. Slowing things down was frequently mentioned:

*“Taking it at a pace where you have the time to genuinely inform people so they’re making informed decisions, creates ownership and creates interest”* (Female non-Aboriginal NGO worker).

For all those interviewed, it was considered critical that families be given all of the information and plenty of time to consider what participation means for their family. It was suggested that researchers need to be really clear about what the goals of the study are and what information will be shared. One of the parents interviewed recognised that for some families, the use of existing data might cause alarm:

*“I guess you’d probably need to explain it to families [in] detail so they don’t get frightened… When you talk about collecting data and things that’s gonna raise alarms…I don’t think there’d be an issue [so long as] the communication level is… spot on”* (Male Aboriginal Parent/Caregiver).

Giving a clear reason why the data is so important and how it will be used to answer important questions for the community was reported as vital. It was clear from the interviews that the researchers have an obligation to go slow and provide a safe space for potential participants to digest the information, to talk with their families and broader networks before rushing into any decision.

### Subtheme (c) tangible benefits for all

Participants felt strongly that any future research must have tangible benefits for the community. The importance of reciprocity for participants was clear:

*“Doing studies and stuff like that, you have to give ‘em back something… ‘cause I mean if families are busy doing all their stuff… just kind of getting by… where’s the… the time and head space… to fill out surveys? … Or to answer questions?” (*Female Aboriginal Parent/Caregiver).

Some of the parents and caregivers shared an expectation of being paid or reimbursed for their time or receiving a gift like a hamper of food. Whilst many parents and caregivers felt optimistic about having the opportunity to share their story, their concerns, their hopes and dreams for their children through the proposed study, some remained sceptical as to whether the proposed research would have any real impact for them. The legacy of past programs that failed to translate findings has, understandably, resulted in scepticism. Stakeholders more often talked about the positives of having longitudinal data on local Aboriginal children such as having clearer findings documented would in turn inform service delivery especially where current data is missing or incomplete.

Making sure that the benefit is spread across the community was also found to be important. This is one of the reasons both stakeholders and parents highlighted the importance of having a “mix of families” in the proposed study representing different language groups from across Central Australia. It was recommended that the inclusion boundary for potential participants be expanded to include the surrounding areas around Alice Springs.

### Subtheme (d) oral stories are important too

We found a general sense that the proposed study would need to give families the opportunity to speak at their pace to say what they really need to say about their story, their situation. A concern was raised that researchers might not be open to really listen, citing past experience of research questions that simply ticked a box. Several participants felt that oral stories were necessary in providing important context around a child’s journey:

*“You could ask not just the numbers, you know, more of the stories, that kind of thing. That’s where these white-fella studies goes wrong”* (Female Aboriginal NGO worker).

Another stakeholder agreed that the numbers collected in reports need some narrative around them to explain the bigger picture.

## Discussion

Ethical guidelines for research with Aboriginal and Torres Strait Islanders repeatedly stress that research must respond to community’s needs and interests. We set out to test the acceptability and feasibility of establishing a longitudinal cohort study of young Aboriginal children in Alice Springs. We spoke with community representatives including parents and caregivers to determine if the idea would be acceptable and to explore which priority issues the study should focus on. We found that participants were positive overall but provided caveats and ideas for how the cohort study should be implemented in a culturally respectful way. Participants acknowledged that it would be useful and interesting. Parents voiced their hypothetical willingness to enrol their children in the study providing sufficient safeguards around data confidentiality and cultural safety were in place. Reference to the need for tangible benefits for the community were consistently highlighted. Our study echoes previous calls for local Aboriginal ownership of research centred on priority issues as determined by the community themselves [21].

Future longitudinal cohort studies should include and track factors such as connection to culture, country, and community from a strengths-based holistic view of Aboriginal health [10]. These protective factors were found to be a great source of strength for Aboriginal communities in Central Australia. Participants also asserted the need to include qualitative storytelling as part of any future study of Aboriginal children and their families. More consideration needs to go into how this could be incorporated to ensure that the burden on families remains low. Future work using Indigenous methodologies could unpack these topics more comprehensively resulting in the inclusion of Indigenous ontologies.

Current funding mechanisms and privacy legislation impede the ability to share data at both an individual and organisational level. This results in poor data resources for organisational planning and families having to retell their stories in each interaction with different service providers. The need for greater collaboration between sectors in the collection and use of data for the benefit of local Aboriginal children was consistently raised by stakeholders. A potential solution suggested to facilitate future longitudinal research included the establishment of a local collaborative network of researchers and service providers. This would complement existing efforts such as the Child Friendly Alice initiative and the important work being done across the NT by Children’s Ground.

It is important to consider both the strengths and limitations of this research. Firstly, a key strength of this study is that it is led by Aboriginal researchers (SE, AH & ES) and conducted in partnership with an Aboriginal controlled health service. AH, ES & SM played a key role in verifying and tailoring our study processes to be culturally responsive and relevant to their local community. We acknowledge that the reported willingness to participate in research in a hypothetical scenario is not an accurate predictor of actual participation [22]. A pilot study would be best placed to assess real world uptake. Therefore, the next phase of this work will involve local Aboriginal researchers and interested community members coming together to lead a co-design team to further explore and design the focus, content and procedures for the future study.

Our study was also limited to the immediate geographical area in and around Alice Springs as serviced by our partners at Congress. We note that the important voices of families living in town camps were not included in this sample. Most of our participants lived in urban areas surrounding the township of Alice Springs and were mature female adults. The results need to be taken within this context. We do not know how well these findings generalise to other geographical locations. But we feel that some of the issues identified are likely to be relevant to other Aboriginal communities in remote areas who share a common history of colonisation. The COVID-19 pandemic extended our timelines further than anticipated. During this time, the NT closed its border to interstate jurisdictions which meant that the Victorian based author (CLJ) needed to conduct many of the interviews with stakeholders via a video conferencing platform. Locally based authors (AH, ES & SM) were able to conduct face-to-face interviews. Rather than accept a reduced sample size we decided to extend the data collection period to overcome these disruptions. Despite these limitations, our study was conducted in a rigorous manner resulting in a large data set of over > 32 h of audio recorded material. We were able to include the views and perspectives of a broad range of participants from teachers, doctors, allied health professionals, early childhood educators, Aboriginal health workers, mothers, fathers, aunties, uncles, and grandparents. We acknowledge that male participants (both stakeholders and parents/caregivers) were an underrepresented subgroup (n = 6). We did not capture the views of fellow researchers’ regarding acceptability and feasibility as the concept, as this will be the focus of the next phase of our research.

## Conclusion

This study has shown that, with a number of important processes in place, the proposed longitudinal cohort study of Aboriginal children is both feasible and acceptable. Participants agreed that any future research must be conducted with strong Aboriginal leadership and involve greater intersectoral collaboration. Furthermore, we established that research must be conducted respectfully by trusted key individuals and at a pace in keeping with the community. It was also noted that the study should be representative of all communities so as to ensure universal benefits from the research. It is our hope that these insights also assist other researchers working on future research plans in partnership with Aboriginal communities in Central Australia. Our paper reinforces existing recommendations of the importance of Aboriginal leadership, the need for clear and transparent communication with research participants and priority driven research that benefits those with whom the research will take place.

### Electronic supplementary material

Below is the link to the electronic supplementary material.

Supplementary Material 1

Supplementary Material 2

Supplementary Material 3

Supplementary Material 4

## Data Availability

Data are available from the authors upon reasonable request and with permission of the Central Australian Aboriginal Congress Research Sub-Committee via the corresponding author.
