# Supplementary material for: “It’s gotta be done right way”: a qualitative study exploring the acceptability of a proposed longitudinal cohort study of young Aboriginal children in Alice Springs
Source: BMC Health Serv Res. 2023 Nov 6;23:1210. doi: 10.1186/s12913-023-10148-9 (PMC10626690; doi:10.1186/s12913-023-10148-9)
Supplement: Supplementary file 2 — Supplementary Material 2 [file 12913_2023_10148_MOESM2_ESM.docx]

# **ADDITIONAL FILE 2**

**“It’s gotta be done right way”: A qualitative study exploring the acceptability of a proposed longitudinal cohort study of young Aboriginal children in Alice Springs**

**POSITIONALITY STATEMENT**

AH, ES & SM have extensive connections within Central Australian Aboriginal communities, as residents, researchers (SM & ES), and Aboriginal health practitioners (AH). These connections facilitated engagement with local stakeholders and Aboriginal parents/caregivers in this project.

AH was born in Alice Springs and has lived most of her life throughout the NT. She is a Yangkunjatjarra woman with familial ties throughout the NT and northern SA.  She works at Congress as the NDIS Support Team Leader and is educated as a social worker. Angela previously worked in the Intensive Family Support Program at Congress providing practical and therapeutic support for vulnerable Aboriginal families for 8 years. Angela led fieldwork for the Healthy Journey for Kids research project in 2020 and continues on as a co-author and Associate Investigator.

ES is a Pitjantjatjara, Yankunytjatjara, Arabana and Adnyamathanha woman working and living on Arrernte Country in Alice Springs with Central Australian Aboriginal Congress. Emma has more than ten years’ experience working in community services, case management, advocacy, administration and community development roles. She has experience working in a trauma informed way, with organisations that work Malparara Way (side by side) in making things better for Anangu (Aboriginal) people. Emma is new to working in research, but believes that all Aboriginal people are researchers by nature, as they follow a 60,000 year-old framework for sharing Tjukurpa (stories) and knowledge.

SM spent the first 10 years of his life living in a remote Aboriginal community (Ampilatwatja) where his parents were involved in interpreting and translating books and documents into the Alyawarr language. His educational background is in Psychology. SM has been working with community participants across Central Australia and the Top End of the Northern Territory on research relating to drug and alcohol use, youth development and sexual health.

The first author (CLJ) is a non-Aboriginal doctoral researcher who grew up on Wathawurrung country in regional Victoria. CLJ is a mother of two children who has worked on early childhood research since 2005 but only in the past 5 years has worked in the field of Aboriginal health. Before commencing work in Alice Springs, the first author completed several short courses focusing on cross cultural communication, cultural safety, and Aboriginal culture in Central Australia. Cultural oversight and mentorship were provided to CLJ by authors SE, AH & ES. CLJ engaged in continuous two way learning with her fellow co-authors and partners at Congress over the course of the project. Findings of this research have been co-presented (CLJ, AH & ES) at the Lowitja 3rd International Indigenous Health and Wellbeing Conference in Cairns, Australia in June 2023. Prior to working at MCRI, the first author lived and studied in Norway where she undertook a research project with Adivasi tribal communities in rural India. Her more recent research experience has been setting up and coordinating a large population based birth cohort study in Victoria. CLJ works in a team with a policy and equity focus at the Murdoch Children’s Research Institute (MCRI) in Melbourne. She has a specific interest in the potential utility of routinely collected data to answer complex Aboriginal health questions.

SE is a Noongar woman from Southwest WA and the first Aboriginal medical doctor to be awarded a PhD in 2003. SE is an experienced epidemiologist who was recently awarded an OA in 2022 for "distinguished service to medical research, to Indigenous health, and to professional organisations". She leads many epidemiological studies including projects based in Central Australia.

SG & AD are both paediatricians. SG is a professor at the MCRI where her research focuses on investigating, testing, and translating sustainable policy relevant solutions that eliminate inequities for children. AD is an associate professor at the University of Melbourne and has lived and worked in the Northern Territory. Her doctoral work led to the creation of the of the ASQ-TRAK developmental screening tool – the first culturally appropriate tool for use with Australian Aboriginal and Torres Strait Islander children. SG, SE & AD are all based in Melbourne, Victoria, but their research network extends across much of Australia.

Each of these roles aided our collective interpretation of the challenges of conducting longitudinal research and the sociocultural context of Central Australia. SM, AH & ES bring an enhanced awareness of, and sensitivity to issues encountered in the study region. We recognise the privilege our higher education and socioeconomic status afford us. This drives the work we do. Our collective experience and insight shaped the way we viewed the data. Study findings were analysed and discussed in light of this reflexivity.
