# Supplementary material for: “It’s gotta be done right way”: a qualitative study exploring the acceptability of a proposed longitudinal cohort study of young Aboriginal children in Alice Springs
Source: BMC Health Serv Res. 2023 Nov 6;23:1210. doi: 10.1186/s12913-023-10148-9 (PMC10626690; doi:10.1186/s12913-023-10148-9)
Supplement: Supplementary file 4 — Supplementary Material 4 [file 12913_2023_10148_MOESM4_ESM.docx]

# **ADDITIONAL FILE 4**

**“It’s gotta be done right way”: A qualitative study exploring the acceptability of a proposed longitudinal cohort study of young Aboriginal children in Alice Springs**

**CONSOLIDATED CRITERIA FOR STRENGTHENING REPORTING OF HEALTH RESEARCH INVOLVING INDIGENOUS PEOPLES: THE CONSIDER STATEMENT**

Huria, T., Palmer, S.C., Pitama, S. *et al.* Consolidated criteria for strengthening reporting of health research involving indigenous peoples: the CONSIDER statement. *BMC Med Res Methodol* **19**, 173 (2019). https://doi.org/10.1186/s12874-019-0815-8

| **Item Checklist Item** | | **Reported on Page No.** |
| --- | --- | --- |
| Governance | |  |
| 1. | Describe partnership agreements between the research institution and Indigenous-governing organization for the research, (e.g., Informal agreements through to MOU (Memorandum of Understanding) or MOA (Memorandum of Agreement)). | Page 3 |
| 2. | Describe accountability and review mechanisms within the partnership agreement that addresses harm minimization. | Page 6-9 |
| 3. | Specify how the research partnership agreement includes protection of Indigenous intellectual property and knowledge arising from the research, including financial and intellectual benefits generated (e.g., development of traditional medicines for commercial purposes or supporting the Indigenous community to develop commercialization proposals generated from the research). | A Collaborative Research Agreement was drafted between Congress and the Murdoch Children’s Research Institute. This agreement outlined the roles and responsibilities of both parties in working together to complete the project. It also  included protection of Indigenous intellectual property and knowledge arising from the research, including financial and intellectual benefits generated. This agreement was executed by executive representatives from both institutes on the 23rd of April 2020. |
| Prioritization | |  |
| 4. | Explain how the research aims emerged from priorities identified by either Indigenous stakeholders, governing bodies, funders, non-government organization(s), stakeholders, consumers, and empirical evidence | Page 3 |
| Relationships (Indigenous stakeholders/participants and Research team) | |  |
| 5. | Specify measures that adhere and honor Indigenous ethical guidelines, processes, and approvals for all relevant Indigenous stakeholders, recognizing that multiple Indigenous partners may be involved, e.g., Indigenous ethics committee approval, regional/national ethics approval processes. | Pages 5-9, 24 |
| 6. | Report how Indigenous stakeholders were involved in the research processes (i.e., research design, funding, implementation, analysis, dissemination/recruitment). | Pages 5-9, 25 |
| 7. | Describe the expertise of the research team in Indigenous health and research. | Additional File 2 |
| Methodologies | |  |
| 8. | Describe the methodological approach of the research including a rationale of methods used and implication for Indigenous stakeholders, e.g., privacy and confidentiality (individual and collective) | Page 5 |
| 9. | Describe how the research methodology incorporated consideration of the physical, social, economic and cultural environment of the participants and prospective participants. (e.g., impacts of colonization, racism, and social justice). As well as Indigenous worldviews. | Page 7-9, Additional File 2 |
| Participation | |  |
| 10. | Specify how individual and collective consent was sought to conduct future analysis on collected samples and data (e.g., additional secondary analyses; third-parties accessing samples (genetic, tissue, blood) for further analyses). | N/A |
| 11. | Described how the resource demands (current and future) placed on Indigenous participants and communities involved in the research were identified and agreed upon including any resourcing for participation, knowledge, and expertise | Page 12 |
| 12. | Specify how biological tissue and other samples including data were stored, explaining the processes of removal from traditional lands, if done, and of disposal. | A data management plan was devised as part of the Collaborative Research Agreement between Congress & MCRI. Data was held at Congress on password protected files on a locally hosted server that was shared securely with MCRI researchers. |
| Capacity | |  |
| 13. | Explain how the research supported the development and maintenance of Indigenous research capacity (e.g., specific funding of Indigenous researchers). | Page 15, 25 |
| 14. | Discuss how the research team undertook professional development opportunities to develop the capacity to partner with Indigenous stakeholders? | Additional File 2 |
| Analysis and interpretation | |  |
| 15. | Specify how the research analysis and reporting supported critical inquiry and a strength-based approach that was inclusive of Indigenous values. | Pages 8-9 |
| Dissemination | |  |
| 16. | Describe the dissemination of the research findings to relevant Indigenous governing bodies and peoples. | Page 21, Additional File 2 |
| 17. | Discuss the process for knowledge translation and implementation to support Indigenous advancement (e.g., research capacity, policy, investment). | Page 21, Additional File 2 |
